# Viewpoint Invariance of Eye Size Illusion Caused by Eyeshadow

**DOI:** 10.3389/fpsyg.2019.01510

**Published:** 2019-07-05

**Authors:** Hiroyuki Muto, Mayu Ide, Akitoshi Tomita, Kazunori Morikawa

**Affiliations:** School of Human Sciences, Osaka University, Suita, Japan

**Keywords:** visual illusion, viewpoint, face, eye, eyeshadow, assimilation

## Abstract

Previous research found that application of eyeshadow on the upper eyelids induces overestimation of eye size. The present study examined whether or not this eyeshadow illusion is dependent on viewpoint. We created a three-dimensional model of a female face and manipulated the presence/absence of eyeshadow and face orientation around the axis of yaw (Experiment 1) or pitch (Experiment 2) rotation. Using the staircase method, we measured perceived eye size for each face stimulus. Results showed that the eyeshadow illusion occurred regardless of face orientation around axes of both yaw and pitch rotations. Crucially, the illusion’s magnitude did not vary across face orientations; lack of interaction between the illusion’s magnitude and face orientation was confirmed by small values of Bayes factors. These findings ruled out the hypothesis that eyeshadow serves as a depth cue and leads to overestimation of eye size due to size-distance scaling. Alternatively, the present findings suggest that the eyeshadow illusion can be well explained by the assimilation between the eyes and eyeshadow, which also facilitates assimilation between the eyes and eyebrows. Practical implications and the present findings’ generalizability are also discussed.

## Introduction

Visual illusions exemplify discrepancies between perception and physical reality. Traditionally, research on visual illusions has employed artificial or simplified figures such as geometric configurations (e.g., the Müller-Lyer illusion, the Delboeuf illusion). However, visual illusions can also occur in more natural and familiar objects like human bodies (e.g., Morikawa, 2003, 2012, 2017; van der Kamp and Masters, 2008). In particular, scientific interest has recently escalated in visual illusions that occur in human faces (e.g., Abe et al., 2009; Kitaoka, 2012; Xiao et al., 2014; Morikawa et al., 2015; Matsushita et al., 2015a,b; Kobayashi et al., 2017; Kiritani et al., 2017a,b). This new field combining visual illusion and facial perception is expected to have not only theoretical implications but also practical applications because it can suggest how to alter facial appearance by intentionally inducing visual illusions with cosmetics.

An example of such cosmetic illusions can be found in a face with eyeshadow. Eyeshadow is a colored cosmetic product applied to the eyelids or skin surrounding the eyes. By using a psychophysical procedure, Morikawa et al. (2015) demonstrated that an average female face with eyeshadow on the upper eyelids appears to have larger eyes than a face without eyeshadow. This eyeshadow illusion was also confirmed in an experiment employing six women whose faces differed (Matsushita et al., 2015b), and this revealed the eyeshadow illusion’s generalizability across varying facial features.

The present study tests whether eyeshadow’s eye-enlarging effect can be generalized to faces seen from various viewpoints. A change of viewpoint has been repeatedly shown to have strong impact on facial recognition (e.g., Bruce, 1982; O’Toole et al., 1998; Favelle et al., 2007; for reviews, Hancock et al., 2000; Johnston and Edmonds, 2009) and on non-face objects (e.g., Tarr and Pinker, 1989; Edelman and Bülthoff, 1992; Tarr, 1995). Nonetheless, most research on visual illusions in faces has used only a frontal view, ignoring potential effects of viewpoint change on the illusion’s magnitude. In our daily lives, we encounter various views of faces. In other words, our faces are seen from various viewpoints by others. Therefore, knowing whether the eyeshadow illusion is dependent on or independent of facial orientation is helpful when applying the cosmetic illusion’s finding to real situations. For example, if the eyeshadow illusion occurs only for the frontal face, people who hope to make their eyes look larger should face straight at the camera when their portraits are taken.

Confirming the eyeshadow illusion’s viewpoint dependence or independence is also theoretically important. So far, two hypotheses have been proposed to explain the eyeshadow illusion’s mechanism. First, according to the assimilation hypothesis (Morikawa et al., 2015; Matsushita et al., 2015b), eyeshadow causes assimilation between the eyes and eyeshadow in the same way as the Delboeuf illusion, where a circle surrounded by a larger ring appears larger than it really is. Moreover, eyeshadow facilitates assimilation between the eyes and the eyebrows (i.e., enhancing perceptual grouping of the eyes and the eyebrows). In their first experiment, Morikawa et al. (2015) examined effects of the distance between the eyes and the eyebrows, the presence/absence of eyeshadow, and the viewing distance between an observer and a display. They found that eyebrows closer to the eyes and the presence of eyeshadow enlarged perceived eye size. More importantly, they also found that the effect of eyeshadow was enhanced when the eyebrows were distant from the eyes at a viewing distance of 5 m, indicating the modulating effect of eyeshadow on assimilation between the eyes and the eyebrows. Furthermore, their second experiment obtained similar results when concentric circles that constitute the Delboeuf illusion figure replaced the eye and eyebrows, and gradation between the inner and outer circles replaced eyeshadow. These findings were considered evidence for the assimilation account.

Another hypothesis, the size-distance scaling account (Abe et al., 2009), states that eyeshadow serves as a depth cue to concavity around the eyes, thereby extending the subjective distance between the eyes and the observer; this leads to overestimation of eye size due to size-distance scaling. By using a paired comparison method, Abe et al. (2009) showed that thicker eyeshadow enhanced the eyes’ subjective depth and enlarged their subjective size. As Matsushita et al. (2015b) and Morikawa et al. (2015) argued, size-distance scaling alone insufficiently explains the eye size illusion’s magnitude (e.g., for an overestimation of 5% at a viewing distance of 60 cm, the eyes should be perceived to be 3 cm back, but this is anatomically impossible). Nonetheless, it is possible that, at least partially, size-distance scaling contributes to the eyeshadow illusion.

The present experiments were designed to determine whether size-distance scaling causes the eyeshadow illusion or the assimilation account alone can fully explain it. In general, illusory depth perception based on pictorial cues is vulnerable to viewpoint changes because changing viewpoints reveal an object’s actual depth information and then attenuate illusory depth. For example, the Ames room illusion occurs only when the room is observed from the ideal viewpoint. Likewise, the actual depth of a face including the hollows around the eyes is more precisely perceived when the face is seen from non-frontal than from frontal viewpoints (e.g., Hill et al., 1997; Ansari and Abdel-Mottaleb, 2005). Therefore, if size-distance scaling contributes to the eyeshadow illusion, then the illusion’s magnitude should decrease as the face turns away from the frontal view. On the other hand, if size-distance scaling is not a major cause of the eyeshadow illusion, then the illusion’s magnitude should remain constant regardless of face orientations. Manipulation of face orientation allows us to test these alternatives.

Confirming our prediction requires decisions about null hypotheses (i.e., evidence for *no* modulating effect of face orientation on the eyeshadow illusion). However, the conventional significance test’s framework does not allow us to support null hypotheses (e.g., Aczel et al., 2018; Dienes and McLatchie, 2018). To overcome this limitation, we conducted Bayes factor (BF) analyses in addition to conventional statistical testing (for a recent review, see Dienes and McLatchie, 2018). In the present context, the BF indicates the extent to which obtained data support an alternative hypothesis over a null hypothesis. The BF value ranges from 0 (in favor of the null hypothesis) through 1 (inconclusive), to infinity (in favor of the alternative hypothesis). According to Jeffrey’s criterion of BF (Jeffreys, 1961; see also Aczel et al., 2018; Dienes and McLatchie, 2018), values from 1/3 to 1/10 are considered substantial evidence and less than 1/10 are strong evidence for the null hypothesis. Additionally, values from 3 to 10 are considered substantial evidence and more than 10 are strong evidence for the alternative hypothesis. Following this criterion, we considered a BF less than 1/3 as evidence for null hypotheses that face orientation does not modulate the eyeshadow illusion (for an example of a similar BF application in research on face perception, see Norman and Tokarev, 2014).

**Figure 1:**
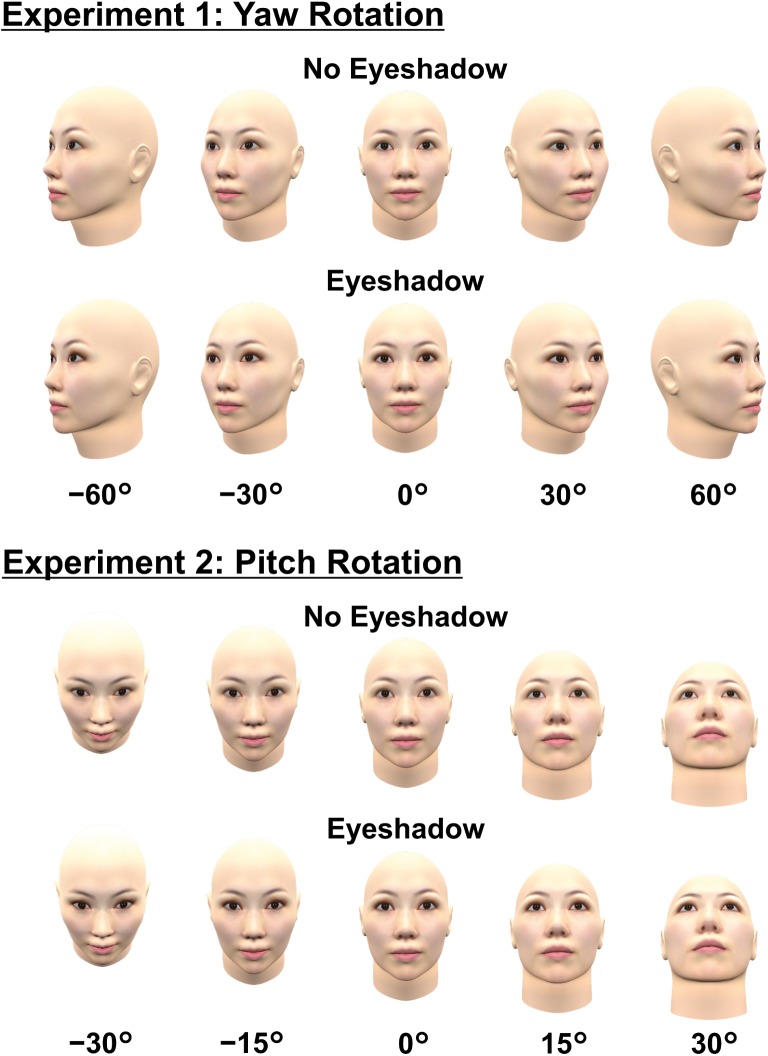
All standard stimuli used in the present experiments.

In summary, the present study examined whether face orientation modulates magnitudes of the eye-enlarging effect induced by eyeshadow. For rigorous manipulations, we created a three-dimensional (3D) model of a female face wearing simulated eyeshadow and manipulated its orientation around the axes of yaw (Experiment 1) and pitch (Experiment 2) rotations (see Figure 1). We measured perceived eye size while the face was viewed from various angles using the staircase method (also known as the up-and-down method), which is a psychophysical procedure.

## Experiment 1

Experiment 1 measured the perceived eye size of faces with and without eyeshadow using a method similar to Experiment 1 in Morikawa et al. (2015). The present experiment’s novelty is in manipulating face orientation around the axis of yaw rotation. The experiment examines whether face orientation modulates eyeshadow’s eye-enlarging effect.

### Method

#### Participants

Twenty undergraduate and graduate students (mean age = 22.3 years, ranging from 19 to 30; 9 males and 11 females) voluntarily participated in Experiment 1. All had normal or corrected-to-normal visual acuity and normal trichromatic color vision.

#### Stimuli and Apparatus

A 3D computer graphic of a face was generated by importing frontal and profile views of an average face of eight Japanese women in their 30s into FaceGen Modeler 3.7. The average face was provided by Shiseido Co. Ltd. Application of brown eyeshadow was simulated by editing a texture image of the face using digital photograph editing software PaintShop Pro XI. The same method was used in Morikawa et al. (2015). The face’s 3D model and the texture image were integrated by the 3D computer graphic software Blender 2.71. The final stimulus images of faces viewed from different angles were created by rotating the face by 0°, ±30°, or ±60° around the vertical axis passing through the midpoint between the eyes (minus and plus signs represent clockwise and counterclockwise directions, respectively). We selected this rotational axis in order to minimize changes in eye size in the 2D stimulus images. Rotating the face by ±60° around the vertical axis passing through the head’s center would have moved both eyes far from the observer, making both eyes’ 2D image much smaller compared to that of the frontal face. Thus, we created 10 standard stimuli (the combination of the presence/absence of eyeshadow and five face orientations; see Figure 1). Stimulus size was matched to Japanese female adults’ average head size, so the frontal face was 14.9 cm (552 pixels) wide at the cheekbone level and 22.0 cm (815 pixels) high from the top of the head to the tip of the chin.

**Figure 2:**
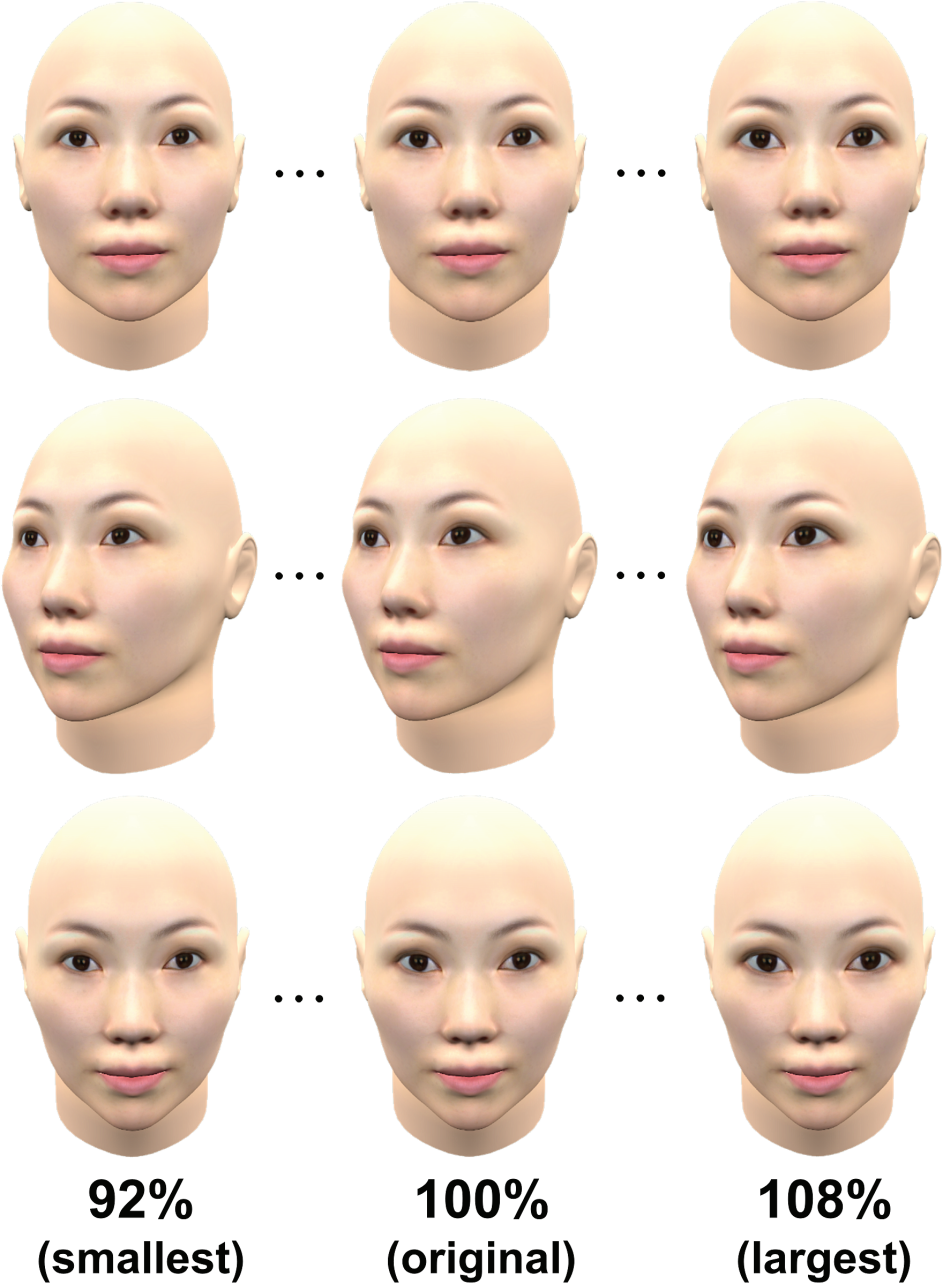
Examples of comparative stimuli used in the present experiments.

As comparative stimuli for the staircase method, we used faces without eyeshadow in all five orientations. Eye size in these stimuli was sequentially reduced or enlarged from 92 to 108% of the original eye size (i.e., 100%) in steps of 1% both horizontally and vertically (Figure 2). To create these comparative stimuli, we reduced or enlarged eye size in the 2D stimulus images (not the 3D model) using PaintShop Pro XI.

All stimuli were presented on a 24.1 inch LCD monitor (NEC MultiSync LCD-PA241W; resolution of 1,920 × 1,200 pixels). The viewing distance between the display and participants’ eyes was about 100 cm.

#### Procedure

Experiments 1 and 2 were carried out in accordance with the recommendations of the research ethics committee of the School of Human Sciences of Osaka University with written informed consent from all participants. All participants gave written informed consent in accordance with the Declaration of Helsinki. The protocol was approved by the research ethics committee of the School of Human Sciences of Osaka University.

The experiment consisted of five blocks for each face orientation^1^. That is, each block was performed to measure the perceived eye sizes of faces with and without eyeshadow in a certain orientation. The blocks’ order was randomized across participants.

**Figure 3:**
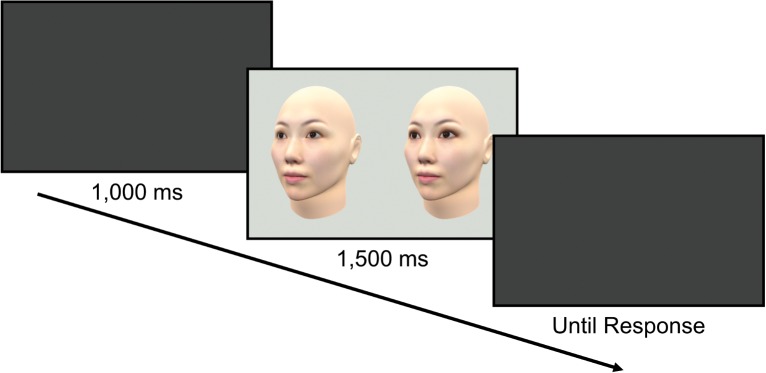
Experimental procedure in the present experiments. A pair of stimuli consisting of the standard and a comparison stimulus were presented side by side. Participants judged which face appeared to have larger eyes.

Figure 3 shows the experimental procedure. Each trial began with a blank gray screen for 1,000 ms. Then, a standard stimulus and a comparative stimulus were displayed side by side on a gray background with a median red, green, and blue (RGB) of 214, 219, and 213. The two faces were always in the same orientation. Participants were asked to judge which face appeared to have larger eyes by pressing the “z” key for the left face or the “backslash” key for the right face. Participants could move their eyes freely to compare the two faces. They were instructed not to focus on a particular point of the stimulus, but to pay attention to the whole face, though their eye gaze was not monitored. The stimulus pair’s presentation lasted for 1,500 ms, followed by a blank gray screen. After the response, the next trial started. We used the staircase method to measure the eye size of the comparative stimulus that was perceived to be the same as that of the standard stimulus. An ascending staircase and a descending staircase for both eyeshadow and no eyeshadow conditions were randomly interleaved; the eye size of the comparative stimulus for each staircase started from either 92% (ascending staircase) or 108% (descending staircase). Thus, each block consisted of four concurrent staircases of trials (i.e., ascending or descending, with or without eyeshadow), which were randomly interleaved. Each staircase was terminated when the direction of the staircase was reversed six times. The left/right position of the standard stimulus and comparative stimulus (i.e., which was presented on the left side) was determined randomly in each trial. The experiment took approximately 20 min on average.

#### Data Analysis

We computed the point of subjective equality (PSE) for each standard stimulus and each participant by averaging eye sizes of the comparative stimuli at which the staircase direction reversed from upward to downward or from downward to upward. Then, we conducted a two-way repeated measures ANOVA with eyeshadow and face orientation as factors. To guard against violations of sphericity, we reported degrees of freedom and *p*-values corrected by Chi-Muller’s ε.

We also calculated BFs by conducting a Bayesian repeated measures ANOVA using JASP 0.8.6.0 (JASP Team, 2018). We used JASP’s default prior distributions (i.e., g-prior distributions with *r* scale values of 1/2 and 1 for fixed and random effects, respectively; for details, see Morey et al., 2018). To calculate BFs for main effects, we used a model with no fixed effect as a null model. For interaction between eyeshadow and face orientation, we used a model containing the two main effects but no interaction as a null model.

### Results and Discussion

**Figure 4:**
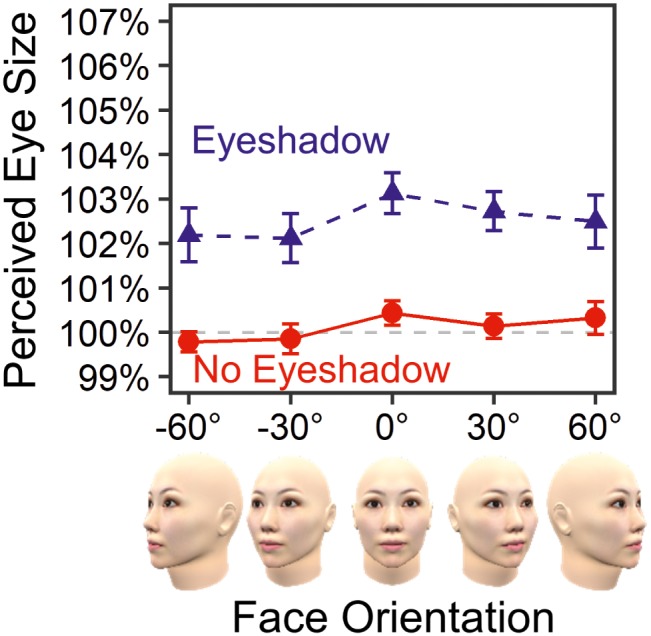
Means and standard errors of perceived eye sizes in Experiment 1.

Figure 4 shows mean PSEs for each standard stimulus. The result confirmed that eyeshadow enlarged perceived eye size by 2.42% on average, *F*(1, 19) = 35.45, $\eta_{p}^{2}$ = .651, *p* < .001, *ε* = 1.00, BF = 8.14 × 10^15^, in favor of the alternative hypothesis. No main effect of face orientation was found, *F*(4, 76) = 1.68, $\eta_{p}^{2}$ = .081, *p* = .163, *ε* = 1.00, BF = 0.07, in favor of the null hypothesis. Most importantly, there was no interaction between eyeshadow and face orientation, *F*(4, 76) = 0.23, $\eta_{p}^{2}$ = .012, *p* = .918, *ε* = 1.00, BF = 0.05, in favor of the null hypothesis. The small BF of 0.05 (<1/3) substantially evidenced lack of face orientation’s modulating effect on the eyeshadow illusion.

By using a 3D computer graphic model, Experiment 1 replicated previous findings on the eyeshadow illusion (Morikawa et al., 2015; Matsushita et al., 2015b). Furthermore, the present experiment found that this illusion is independent of face orientation at least around the axis of yaw rotation. In the next experiment, we examine whether the finding of viewpoint independence can also be observed for a face rotated around its pitch axis.

## Experiment 2

Manipulations of face orientation around varied rotational axes might have different consequences (this is the case for face recognition; see Favelle et al., 2007). Since eyeshadow (like the eyebrows) is usually placed above the eyes, a face’s pitch rotation reduces vertical distances among the eyes, the eyebrows, and eyeshadow on the two-dimensional retinal image. In contrast, yaw rotation less affects vertical distances among them. Given that the eyebrows’ position affects the eyes’ perceived size and shape (Morikawa et al., 2015; Matsushita et al., 2015a) and that the magnitude of the Delboeuf illusion is a function of a diameter ratio between the two circles (e.g., Oyama, 1960, 1962; Goto et al., 2007), the retinal image’s distortion caused by rotation potentially affects the eyeshadow illusion’s magnitude, independent of perceived depth. Alternatively, it is also possible that the eyeshadow illusion is based on the real shape of a face (i.e., the frontal face) rather than the retinal image, which may be explained by shape constancy. To address this issue, Experiment 2 employed the same procedure as Experiment 1, except for manipulation of face orientation on the axis of pitch rotation instead of yaw rotation.

### Method

#### Participants

Twenty undergraduate and graduate students (mean age = 22.8 years, ranging from 20 to 28; 9 males and 11 females) voluntarily participated in Experiment 2. All had normal or corrected-to-normal visual acuity and normal trichromatic color vision. None had participated in Experiment 1.

#### Stimuli

We used the same model and texture as in Experiment 1. Instead of yaw rotation, we rotated the face’s frontal view by 0°, ±15°, or ±30° around the horizontal axis passing through the midpoint between the eyes (minus and plus signs represent downward and upward directions, respectively). We selected this axis so as to minimize changes in eye size in 2D stimulus images. We set the range of rotational angles narrower than in Experiment 1 (i.e., ±60°) because the eyes are horizontally long and more likely to be obscured by pitch than by yaw rotation at the same angle. As in Experiment 1, we created a total of 10 standard stimuli (the combination of the presence/absence of eyeshadow and five face orientations; see Figure 1). Comparative stimuli for the staircase method were created by sequentially changing the eye size in stimuli without eyeshadow, just in the same way as in Experiment 1 (see Figure 2).

#### Apparatus, Procedure, and Data Analysis

The apparatus and the experimental and statistical procedures were the same as in Experiment 1. The experiment took approximately 20 min.

### Results and Discussion

**Figure 5:**
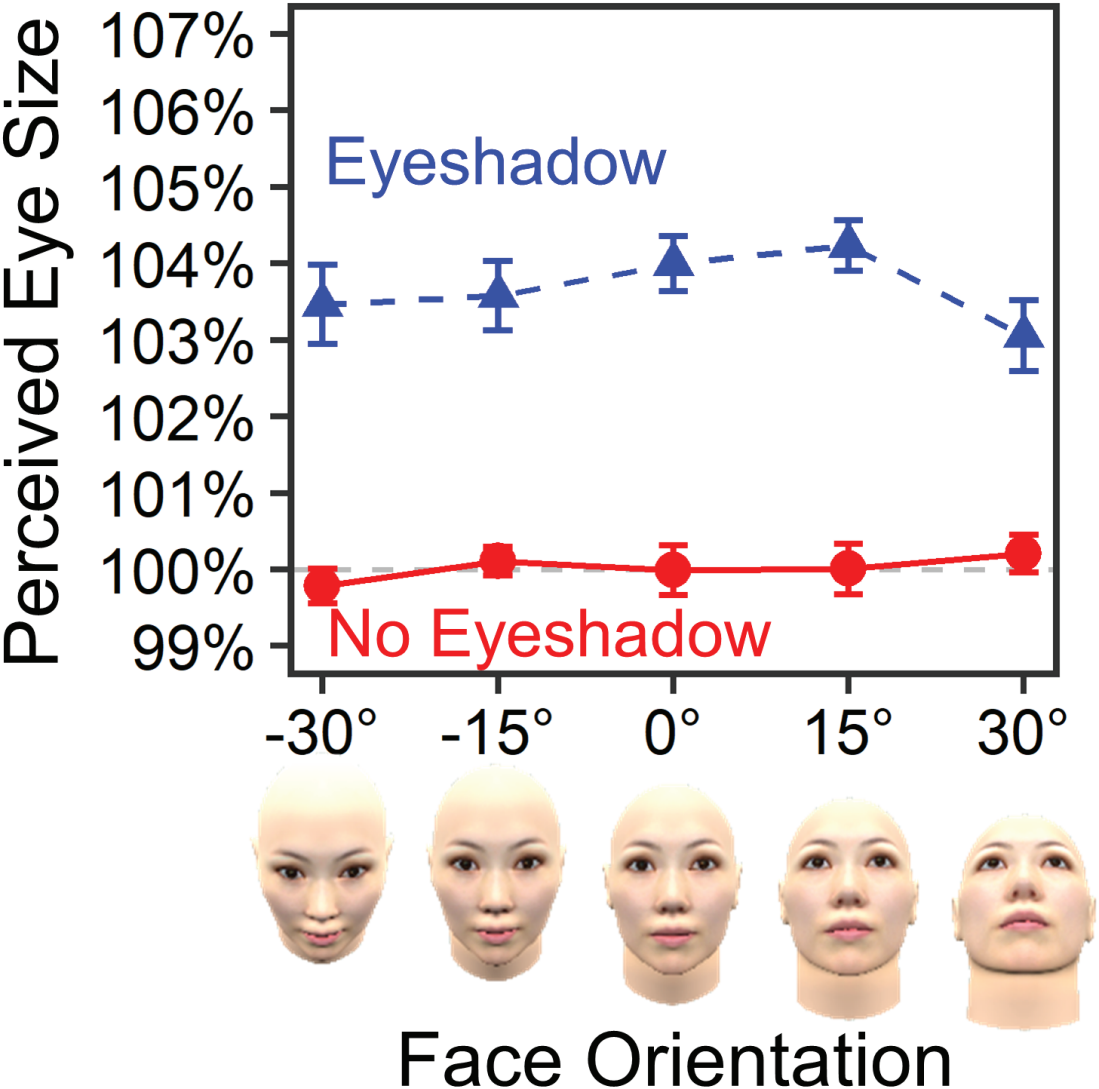
Means and standard errors of perceived eye sizes in Experiment 2.

Figure 5 shows mean PSEs for each standard stimulus. Again, the result showed the eye-enlarging effect caused by eyeshadow (3.65% on average), *F*(1, 19) = 126.89, $\eta_{p}^{2}$ = .870, *p* < .001, *ε* = 1.00, BF = 3.64 × 10^36^, in favor of the alternative hypothesis. No main effect of face orientation was found, *F*(3.65, 69.31) = 0.92, $\eta_{p}^{2}$ = .046, *p* = .451, *ε* = .91, BF = 0.02, in favor of the null hypothesis. Most importantly, there was no interaction between eyeshadow and face orientation, *F*(3.80, 72.18) = 1.40, $\eta_{p}^{2}$ = .069, *p* = .243, *ε* = .95, BF = 0.19, in favor of the null hypothesis. The small BF of 0.19 (<1/3) substantially evidenced lack of modulating effect of face orientation around the pitch axis in the eyeshadow illusion.

In summary, Experiment 2 revealed that the eyeshadow illusion was independent of face orientation around the axis of pitch rotation. This finding is similar to that on yaw rotation in Experiment 1.

## General Discussion

By means of rotated faces created from a 3D computer graphic model, these two experiments examined whether the eye-enlarging illusion caused by eyeshadow is dependent on or independent of viewpoint. We confirmed that the eyeshadow illusion occurred for a 3D computer graphic model, replicating and extending previous research using an average female face (Morikawa et al., 2015) and photographs of female models (Matsushita et al., 2015b). More importantly, our results consistently demonstrated that the eyeshadow illusion’s magnitude was constant across face orientations around the yaw (Experiment 1) and pitch (Experiment 2) rotations, as evidenced by BF analyses. Thus, the present study provides evidence for viewpoint invariance of the eyeshadow illusion.

Theoretically, the present findings suggest that size-distance scaling does not contribute to the eyeshadow illusion. As explained in the section “Introduction,” a non-frontal viewpoint should reveal actual depth information of the eye area and thus attenuate illusory depth perception caused by a pictorial cue that is most effective when seen from the frontal viewpoint. Given this, if the size-distance scaling account (Abe et al., 2009) were true, then the eyeshadow illusion’s magnitude should have decreased as the face turned away from its frontal view. Therefore, taken together with Matsushita et al. (2015b) and Morikawa et al. (2015), the eyeshadow illusion can be well explained by the assimilation account alone. This account is consistent with the notion that illusions occurring in the human body and face are likely to be assimilative rather than contrastive, due to biological co-occurrences of similar characteristics throughout the body and face (Morikawa, 2012, 2017). However, the present study did not obtain direct evidence supporting the assimilation account. More studies are needed to examine the contribution of the assimilative effect and clarify in more detail the mechanisms underlying the eyeshadow illusion.

Furthermore, the present study does not explain the mechanisms of the Delboeuf illusion and the assimilative effect. Although this topic is outside the scope of the present article, one promising candidate for such mechanisms is grouping of low-level image features at some spatial frequency bands. The image analysis approach with sub-band decomposition with Laplacian pyramid could be a fruitful method for future research toward an explanation for why eyeshadow makes eyes appear larger.

Manipulation of face orientation around the axis of pitch rotation, which has been much less studied than yaw rotation, is also a novel point of the present study. By pitch rotation, unlike yaw rotation, the vertical distances among the eyes, the eyebrows, and eyeshadow on the two-dimensional retinal image are changed. Indeed, in Experiment 2, the distance between the top of the palpebral fissure and the lower edge of the eyebrow was approximately 16 mm (45′) for the face at 0° or 13 mm (56′) at ±30° on the vertical line that passed through the pupil’s center. Nonetheless, face orientation around axes of pitch or yaw rotation did not affect the eyeshadow illusion’s magnitude. This finding suggests that the eyeshadow illusion occurring in rotated faces cannot be fully explained by low-level image features in the 2D image because the Delboeuf illusion is known to be sensitive to the distance between circles (Morikawa et al., 2015). We speculate that the present finding of viewpoint invariance might be accounted for by shape constancy of faces viewed from different angles. That is, observers could perceive a rotated face in the same way as from the frontal viewpoint, regardless of slight distortion of their retinal images, at least within the angular range tested in the present experiments. To test this speculation, future research should measure the magnitude of the eyeshadow illusion or other related illusions (e.g., the Delboeuf illusion) using stimuli wherein shape constancy is violated (e.g., by using slanted pictures; e.g., Hanada, 2005). It is also useful to systematically manipulate vertical distances among the eyes, the eyebrows, and eyeshadow as well as face orientations, across a range broader than in the present study. Furthermore, although we used the horizontal axis passing through the midpoint between the eyes for pitch rotation to minimize changes in eye size in the 2D stimulus images, this axis was somewhat unnatural. Thus, it may be informative if future studies examine whether and how the selection of a rotational axis affects face perception and illusions.

The present findings also have practical implications for makeup techniques given that the eyes are an important determinant of facial attractiveness. Specifically, larger eyes are known to make female faces more attractive (Geldart et al., 1999; Baudouin and Tiberghien, 2004). Previous studies suggested that an illusory overestimation of eye size also led to enhanced attractiveness (Morikawa et al., 2015; Matsushita et al., 2015a). In addition, strong correlations exist between frontal and lateral facial attractiveness (Rule et al., 2009; Gu et al., 2018). Given these findings, although we did not measure faces’ attractiveness, the present findings suggest that eyeshadow possibly makes female faces more attractive via overestimation of eye size regardless of facial orientation.

The present study has some limitations. First, since we used a 3D model to create facial stimuli for rigorous manipulations, we should be cautious about whether the eyeshadow illusion’s viewpoint independence can be observed in real human faces. Second, whether the eyeshadow illusion and its viewpoint independence are linked to holistic processing specific to face perception still remains unclear (e.g., Tanaka and Farah, 1993; Hayward et al., 2016). So far, the eyeshadow illusion has been observed for an average of Japanese female faces (Morikawa et al., 2015) and photographs of real Japanese female models (Matsushita et al., 2015b). Moreover, a similar effect was found even for the Delboeuf illusion with gradation that simulated eyeshadow (Morikawa et al., 2015). Taking these observations into account, that general visual processing for various objects—not limited to faces—drives the eyeshadow illusion seems more plausible; then, the present finding on viewpoint independence could be generalized to stimuli other than faces. Nonetheless, it is possible that the present findings depend on the 3D facial structure of a particular ethnicity, age, and gender that we used. For example, it remains to be seen whether the viewpoint invariance of the eyeshadow illusion also applies to Caucasian faces that have deeper eye sockets than Asian faces. To draw a stronger conclusion, future research should apply similar viewpoint manipulation to photographs of real faces including a variety of races, ages, and genders, and inverted faces. It would also be helpful to use non-face objects such as spheres on which the Delboeuf illusion figure is drawn.

Although we used only brown eyeshadow, the color of eyeshadow might also influence the eyeshadow illusion or other aspects of facial impression. In particular, the combination between colors of eyeshadow and facial skin possibly modulates the assimilation effect. For example, eyeshadow whose color is quite different from skin color might be perceived as a feature independent of a face; in turn, this might decrease the assimilation effect. In addition, given that skin color is assimilated with eyeshadow color (Kiritani et al., 2017a) and lip color (Kobayashi et al., 2017; Kiritani et al., 2017b), dynamics between multiple facial features should be taken into account.

Further research is needed to determine whether other illusions occurring in faces are also, like the eyeshadow illusion, viewpoint independent. For example, does a viewpoint change weaken the chromatic assimilation between skin color and lip color (Kobayashi et al., 2017; Kiritani et al., 2017b)? Since the area of the retinal image of colored facial parts (e.g., eyeshadow, the lips) becomes smaller as the face rotates away from its frontal view, such color assimilation could be attenuated by the change in viewpoint. On the other hand, given the biological co-occurrence hypothesis (Morikawa, 2012, 2017) and shape constancy, such assimilation could continue to work if at least a small portion of colored facial features can be observed. To further understand the mechanism of biological illusions and to confirm whether cosmetic illusions can be applied to more natural situations, the present approach of manipulating face orientation is useful.

## Data Availability

The raw data supporting the conclusions of this manuscript will be made available by the authors, without undue reservation, to any qualified researcher.

## Ethics Statement

Experiments 1 and 2 were carried out in accordance with the recommendations of the research ethics committee of the School of Human Sciences of Osaka University with written informed consent from all participants. All participants gave written informed consent in accordance with the Declaration of Helsinki. The protocol was approved by the research ethics committee of the School of Human Sciences of Osaka University.

## Author Contributions

HM, MI, and KM conceived and designed the experiments. HM and MI created stimuli. HM collected and analyzed the data and drafted the paper. AT and KM provided critical revision. All authors have approved this version of the manuscript and its submission.

### Conflict of Interest Statement

The authors declare that the research was conducted in the absence of any commercial or financial relationships that could be construed as a potential conflict of interest.

## Footnotes

^1^Instead of randomly interleaving all orientation conditions within a session, we blocked trials by face orientation to eliminate participants’ unpredictability of face orientation. The adoption of the block design may have eased participants’ judgment and thus allowed more precise measurement of perceived eye size.
